# Post-segregational Killing and Phage Inhibition Are Not Mediated by Cell Death Through Toxin/Antitoxin Systems

**DOI:** 10.3389/fmicb.2018.00814

**Published:** 2018-04-25

**Authors:** Sooyeon Song, Thomas K. Wood

**Affiliations:** Department of Chemical Engineering, Pennsylvania State University, University Park, PA, United States

**Keywords:** toxin/antitoxin systems, persistence, growth inhibition, biofilm, stress response

## Review

We recently elucidated several misperceptions in a field related to toxin/antitoxin (TA) systems, persister cell biology (Kim and Wood, 2016, 2017). Here, in the following sections, we aim to initiate a similar discussion about several inaccurate yet entrenched theories that are related to TA systems. Most of these misperceptions are based on overproducing toxins; i.e., many toxins of TA systems appear lethal if produced from a strong promoter. However, conclusions based on overproducing toxins may not be relevant for physiological conditions.

The toxin/antitoxin (TA) field, born of controversy, is plagued by several prevailing misperceptions. For example, some TA systems stabilize plasmids and other genomic regions; however, the evidence of post-segregational killing by toxins of TA systems is weak. In addition, the evidence is weak for cell death via TA systems like MazF/MazE and weak for TA systems mediating death in phage exclusion systems. Although many aspects of the biological roles of TA systems remain enigmatic, there are now some clear, confirmed TA functions: (i) phage inhibition, (ii) plasmid maintenance, (iii) stress response (including regulation of loci distinct from the TA pair itself), (iv) biofilm formation, and (v) persistence. Therefore, this opinion piece aims to challenge the oft-repeated claims of cell killing related to TA systems with the goal of emphasizing their primary biological role: constraining metabolism in a reversible manner. Hence, their roles in their five confirmed functions all stem from their ability to rapidly and reversibly reduce metabolic activity.

### Nebulous nomenclature

Historically, TA systems were deemed cytosolic (not secreted); hence, TA system toxins were considered primarily to affect the metabolism of the host that produced the TA system rather than as serving as a weapon against other cells, such as colicins (Rendueles et al., 2014). With a better understanding of their biological function, growth diminution as opposed to cell death, TA systems would be more aptly named “growth inhibitors” and “silencers of growth inhibitors,” rather than “toxins,” which imply a poison used against competitors rather than an internal means to reduce metabolism. However, little would be served, and much confusion would ensue, if the name of the field was changed now.

Moreover, TA system components are starting to be identified outside the cell. For example, the type VII secretion system DNase toxin EsaD of the EsaD/EsaG TA system of *Staphylococcus aureus* (Cao et al., 2016) is secreted for bacterial competitiveness; this is the second known toxin that is a DNase with RalR of the *Escherichia coli* RalR/RalA TA system being the first (Guo et al., 2014). The *Xanthomonas oryzae* type III secretion system toxin AvrRxo1 of the AvrRxo1/Arc1 TA system is also secreted into plants and modifies nicotinamide adenine dinucleotide (Shidore et al., 2017). Additionally, antitoxin MqsA of the MqsR/MqsA TA system of *Xylella fastidiosa* (Santiago et al., 2016) is secreted via outer membrane vesicles. Therefore, the distinction between external toxins like colicins and TA systems is becoming less clear.

Note that for TA pairs, we prefer the convention of listing the toxin first, then the antitoxin (e.g., MazF/MazE), regardless of the gene order. In addition, we prefer naming the antitoxin with four letters ending with an “A” (e.g., MqsA), where possible, to more readily identify the antitoxin.

How TA systems are classified is also evolving and is based on the mechanisms by with antitoxins mask toxin activity. There are six well-established systems (Page and Peti, 2016) that include type I (e.g., Hok/Sok), in which the antitoxin RNA prevents toxin mRNA translation (Gerdes et al., 1986a), type II (e.g., CcdB/CcdA), in which the antitoxin protein binds directly to the toxin to inhibit it (Ogura and Hiraga, 1983), type III (e.g., ToxN/ToxI), in which the antitoxin RNA binds the toxin to inhibit it (Fineran et al., 2009), type IV (e.g., CbtA/CbeA), in which the antitoxin protein interferes with the binding of the toxin with its target (Masuda et al., 2012), type V (e.g., GhoT/GhoS), in which the protein antitoxin is an RNase that cleaves specifically the toxin mRNA (Wang et al., 2012), and type VI (e.g., SocB/SocA), in which the antitoxin protein causes the degradation of the toxin (Aakre et al., 2013). A type VII system (e.g., Hha/TomB) has also recently been discovered in which the antitoxin is an enzyme, but rather than cleaving toxin mRNA as in the type V system, the type VII antitoxin inactivates the toxin by oxidizing a cysteine residue (Marimon et al., 2016).

### Plasmid stabilization is not based on post-segregational killing

Toxin/antitoxin systems are ubiquitous in that they are found in nearly all prokaryotic and archaeal genomes (Pandey and Gerdes, 2005). The first TA system, CcdB/CcdA (coupled cell division), was discovered in 1983, and was shown to maintain a mini-F plasmid (Ogura and Hiraga, 1983). In this seminal work, there was no mention of post-segregational killing or cell death, and toxin CcdB was shown to inhibit host cell division (Ogura and Hiraga, 1983).

The genesis of the idea of post-segregational killing arose with the report of the second TA system, Hok/Sok (*h*ost *k**illing* and *s*uppressor of *k**illing*), that also stabilized a plasmid (R1) (Gerdes et al., 1986b). In this report, that includes “Post-segregational killing of plasmid-free cells” in the title, the authors indicated, “we propose that the parB+ locus mediates plasmid stability by killing cells that have lost the *parB*+ plasmid… ” and claimed “irreversible killing of the host.” Unfortunately, little evidence was presented of cell death with physiologically-relevant levels of Hok toxin; in fact, the authors found a lack of cell lysis as demonstrated by a lack of extracellular β-galactosidase activity and only saw a decrease in cell viability when Hok toxin was produced by the strong lambda promoter (Gerdes et al., 1986b). Furthermore, ghost cells were produced (but not a reduction in cell viability) with a copy number 8 plasmid construct harboring a cloned *hok/sok* locus (presumably under wild-type regulation but this is not clear), and R1 plasmids (the physiological host harboring *hok/sok*) have a copy number of 6 (Ehrenberg and Sverredal, 1995). Later reports also lack cell killing with physiological-relevant amounts of Hok; i.e., killing is shown via overproduction of Hok (Gerdes et al., 1986a). Hence, although some TA systems clearly stabilize plasmids, cell killing of plasmid-free hosts has not been demonstrated.

### MazF does not cause programmed cell death

As a corollary to the lack of evidence for cell death in plasmid maintenance studies outlined in the previous section, data indicating toxin MazF of the *E. coli* MazF/MazE TA system is responsible apoptotic behavior (i.e., programmed cell death, PCD) are also suspect. For example, although roughly 80% of vegetative *Myxococcus xanthus* cells undergo PCD during nutrient-limiting development (one of the few examples of bacterial PCD), it was claimed that MazF mediated this killing (Nariya and Inouye, 2008). However, this result was later shown to be an artifact of a defect in the PilQ1 secretin of this strain used in the initial report by two different groups (Lee et al., 2012; Boynton et al., 2013). Hence, PCD does not occur for wild-type *M. xanthus* strains through MazF.

Similarly, it is doubtful that MazF is responsible for PCD in *E. coli*. PCD was claimed in 1996 for *E. coli* MazF (Aizenman et al., 1996) and has been repeated frequently (Engelberg-Kulka and Glaser, 1999; Sat et al., 2001; Zhang et al., 2003; Kumar et al., 2016). Unfortunately, the initial claim of cell death was based on overproduction of MazF via a lambda P_L_ promoter (Aizenman et al., 1996). Furthermore, later studies from independent labs show PCD does not occur (Pedersen et al., 2002; Tsilibaris et al., 2007). Instead, MazF induces a reversible inhibition of growth (Pedersen et al., 2002). Therefore, MazF at physiological levels does not cause PCD in any organism to date (Boynton et al., 2013).

### The extracellular death factor is unlikely to mediate cell death through MazF

The extracellular death factor (EDF, Asn-Asn-Trp-Asn-Asn) has been proposed as a quorum-sensing element for *E. coli* that mediates PCD via MazF (Kolodkin-Gal et al., 2007). Unfortunately, it appears far more likely that this pentapeptide simply interacts with antibiotics extracellularly and has little to do with PCD or quorum sensing. Critically, EDF has been shown to scavenge free radicals (Gao et al., 2010) and to protect cells from ampicillin addition (Yan et al., 2015). One of the main problems with the claim of quorum sensing via EDF is the necessity of adding rifampicin to see an effect (Kolodkin-Gal et al., 2007); however, quorum-sensing systems do not, in general, require antibiotic pre-treatments to function, and the addition of rifampicin to inhibit transcription should activate many of the type I TA systems in *E. coli* in which the antitoxins are labile. Furthermore, no genes have been shown to be controlled by EDF nor has a protein import or export system been elucidated; yet, quorum-sensing systems are based on secreted signals being either recognized by surface proteins or being imported and then affecting gene expression, like AI-2 (Pereira et al., 2013) and indole (Lee et al., 2015) in *E. coli*. Also, the protein from which EDF is supposed to be derived, Zwf, contains the sequence NNWDN, not NNWNN (Kumar et al., 2016). Hence, many aspects of a quorum-sensing system have never been demonstrated for EDF. Since the previous section outlines why MazF does not cause PCD, by the transitive property, it is doubtful that EDF mediates PCD through MazF (if PCD does not exist via MazF).

### TA systems do not inhibit phage through altruistic killing

For the similar misperception that TA systems are involved in cell killing during phage inhibition, we must shoulder some of the blame. We first linked TA systems to phage propagation by discovering that the *E. coli* type I TA system Hok/Sok inhibits T4 phage propagation (Pecota and Wood, 1996); unfortunately, we also suggested the mechanism included altruistic killing. The role of TA systems in inhibiting phage propagation was verified 8 years later using the type II MazF/MazE TA system to show a reduction in P1 phage propagation (Hazan and Engelberg-Kulka, 2004) and 13 years later with the type III ToxN/ToxI TA system (Fineran et al., 2009). Phage inhibition has also been reported for a likely type IV system, AbiEii/AbiEi from *Lactococcus lactis* (Dy et al., 2014). Therefore, phage inhibition has been shown for the four best-characterized types of TA systems. Additional proof of the role of TA systems in phage inhibition stems from the fact that phage carry antitoxins to combat host-encoded toxins (Otsuka and Yonesaki, 2012), as well as encode protease inhibitors to prevent activation of type II TA systems (Sberro et al., 2013). So the role of TA systems in inhibiting phage propagation is solid.

However, in all these cases, cell death was not shown with a physiological dose of toxin. For example, for ToxN/ToxI, even with a pBad promoter for toxin induction in *E. coli*, the authors found the TA system did not cause cell lysis and was reversible (Fineran et al., 2009); yet, this became apoptotic and beneficial behavior in later publications: “abortive infection, during which a bacteriophage-infected cell altruistically commits suicide to protect the clonal population, can be mediated by a TA pair” (Blower et al., 2011). This misstatement was based on two references that do not indicate cell suicide but instead show growth inhibition (Blower et al., 2009; Fineran et al., 2009). To be clear, there may be some altruistic behavior in abortive infection systems based on other proteins, but cell death has not been shown to be mediated by TA systems. Therefore, it is most likely that the decrease in growth rate mediated by toxins (activated by phage attack) leads to phage inhibition.

Given this mechanism, one would also surmise that any protein that slows growth would inhibit phage propagation (Wang and Wood, 2011). Hence, what makes TA systems interesting for phage inhibition is how they are induced by phage attack. In our original publication (Pecota and Wood, 1996), we proposed Hok/Sok was induced passively since T4 phage rapidly inhibits host transcription by modifying RNA polymerase, which leads to preferential degradation of the antitoxin RNA (Sok) and thereby activation of toxin Hok.

### Guanosine tetraphosphate (ppGpp) and SpoT are not a TA system

Different from the heretofore undocumented claims of cell death from physiologically-relevant levels of toxins of TA systems, it was proposed that a metabolite, ppGpp, was a “toxin” and SpoT (ppGpp 3′-pyrophosphohydrolase) was an “antitoxin” (Amato et al., 2013). This claim is inaccurate since ppGpp is merely the substrate of SpoT, so if this trend were continued, one could claim any metabolite that slows growth in excess along with a protein that reduces its level as a TA pair. For example, following this logic, indole could be considered a “toxin” since it inhibits growth above 2 mM and AcrEF could be considered an “antitoxin” since it exports indole (Lee et al., 2015). Clearly this not appropriate. Even more perplexing is that ppGpp is not a toxin in that it serves to orchestrate the stringent response. Furthermore, the authors used carbon source transitions to form cells they called “persisters” but this, too, has been shown to be inaccurate (Kim and Wood, 2017). Finally, their claim that cyclic adenosine monophosphate increases persistence (Amato et al., 2013) was also found to be incorrect in that cyclic adenosine monophosphate decreases persistence based on three lines of evidence (Kwan et al., 2015b).

### There are clear biological roles for TA systems, including the stress response

Even this year it has been written that “the biological roles of many TA modules still remain elusive” (Harms et al., 2018). Although strictly true, we feel it is more accurate to indicate there are several clear physiological roles of TA systems. For example, the physiological roles of the MqsR/MqsA TA system are myriad (e.g., biofilm formation, stress response, global gene regulations, and persistence) and linked by oxidative stress. MqsR/MqsA of *E. coli* was discovered in 2004 via a whole-transcriptome study that identified it as the first TA system induced in biofilms (Ren et al., 2004). The structure of the TA pair was used to discern that the toxin MqsR is an RNase (Brown et al., 2009); by cleaving RNA with 5′-GCU sites independent of ribosomes (Yamaguchi et al., 2009), MqsR can rapidly re-direct metabolism. In addition, MqsA was found to not only regulate its own promoter but to control the oxidative stress response (over 500 genes) by controlling sigma factor RpoS by binding to an upstream palindrome (Wang et al., 2011); this result reduces biofilm formation indirectly by decreasing cyclic diguanylate (c-di-GMP) levels (Wang et al., 2011). MqsA also controls biofilm directly by repressing the promoter of the curli regulator CsgD (Soo and Wood, 2013). Hence, MqsA is a global regulator that serves to reduce biofilm formation in the absence of oxidative stress (by repressing *rpoS* and *csgD*).

Oxidative stress is experienced as the commensal strain *E. coli* encounters bile acid, so MqsR/MqsA helps the cell weather oxidative stress in the gastrointestinal tract (Kwan et al., 2015a). Furthermore, the MqsR/MqsA TA system controls TA system GhoT/GhoS by toxin MqsR cleaving preferentially the mRNA of antitoxin GhoS, so during oxidative stress, MqsR activates another TA system as a cascade (Wang et al., 2013); toxin MqsR cleaves all but 14 *E. coli* mRNAs and one of these 14 mRNAs that lacks a 5′-GCU site is GhoT. The result is that cell metabolism is reduced two ways: by (i) eliminating nearly all mRMA via toxin MqsR cleaving 5′-GCU sites and (ii) reducing ATP via toxin GhoT (Cheng et al., 2014). When growth is extremely limited by this MqsR activity, the cell becomes dormant and able to withstand extreme stress as evidenced by the fact that MqsR/MqsA is the first TA system that when deleted, reduces persistence (Kim and Wood, 2010) and MqsR increases persistence through GhoT (Wang et al., 2013). Hence, there is a clear physiological role for the MqsR/MqsA TA system: it conveys the oxidative stress created by bile acid through MqsA degradation which derepresses the stress response of the cell (including increasing biofilm formation) and through MqsR differentially cleaving mRNA and activating GhoT (that leads to persistence in some cells).

This role of antitoxins controlling more than their own promoter was confirmed by antitoxin DinJ of the *E. coli* YafQ/DinJ TA system; DinJ binds and represses the promoter *csgE* (a positive regulator of RpoS mRNA translation) and thereby reduces the translation of the RpoS transcript (Hu et al., 2012). Upon stress, antitoxin DinJ is degraded and RpoS translation is enhanced by CsgE which leads to less motility, an increase in catalase activity, an increase in c-di-GMP, and an increase in biofilm formation. Antitoxins acting as global regulators extends to other strains as well; for example, in *S. aureus*, the SavS/SavR TA system silences the virulence genes *hla* and *efb* by binding their promoters (Wen et al., 2018). Hence, the role of antitoxins as global regulators is established.

In addition, the TisB/IstR-1 type I TA system has been shown to have the physiological role of producing persister cells when the cell is stressed by ciprofloxacin (Dörr et al., 2010). Ciprofloxacin damages DNA which induces TisB toxin via an SOS response which results in TisB injuring the membrane thereby reducing the proton motive force and ATP levels.

### Conditional cooperativity only holds for some TA systems

It is frequently indicated that conditional cooperativity is the prevalent form of type II TA system regulation (Harms et al., 2018). The idea is that antitoxins can exist as dimers and bind more than one toxin. At low ratios of toxin to antitoxin, the TA complex represses the TA promoter more than when the ratio of toxin to antitoxin is high. Although this concept may hold for some TA systems, there seem to be as many exceptions as there are those that fit this model. For example, the binding of MqsR toxin to MqsA causes the complex to fall off DNA since the binding sites of MqsA to DNA and to MqsR overlap, so MqsA cannot bind both its promoter and toxin. Hence, the regulation of *mqsRA* is not governed by conditional cooperativity (Brown et al., 2013). Similarly, the *E. coli* YafQ/DinJ TA system (Ruangprasert et al., 2014), the *Yersinia pestis* HicA3/HicB3 TA system (Bibi-Triki et al., 2014), and the *Proteus vulgaris* HigB-HigA TA system (Schureck et al., 2014) are all not regulated by conditional cooperativity.

## Perspectives

Charles Darwin emphasized the importance of natural selection for beneficial mutations; i.e., changes that lead to improved reproduction will become dominant (Darwin, 1859). For bacteria, this is often oversimplified to imply organisms with fast growth will dominate. However, since most bacterial cells are starving (Schmidt, 2012), it appears it is just as relevant for microbes to elegantly decelerate their growth; i.e., the survival of the fittest doctrine should include selection of organisms with elegant diminution of growth. This elegant growth diminution is the realm of TA systems, where they are clearly utilized to inhibit phage, stabilize genetic elements, activate the stress response, regulate biofilm formation, and generate persister cells. Hence, TA systems are nearly ubiquitous in prokaryotes since they afford means to reduce growth elegantly. However, they are not utilized to kill cells.

## Author contributions

TW conceived and wrote the manuscript. SS helped write the manuscript.

### Conflict of interest statement

The authors declare that the research was conducted in the absence of any commercial or financial relationships that could be construed as a potential conflict of interest.

## References

[B1] Aakre C. D. Phung T. N. Huang D. Laub M. T. (2013). A bacterial toxin inhibits DNA replication elongation through a direct interaction with the β sliding clamp. Mol. Cell 52, 617–628. 10.1016/j.molcel.2013.10.014 24239291 PMC3918436

[B2] Aizenman E. Engelberg-Kulka H. Glaser G. (1996). An *Escherichia coli* chromosomal “addiction module” regulated by guanosine 3',5'-bispyrophosphate: a model for programmed bacterial cell death. Proc. Natl. Acad. Sci. U.S.A. 93, 6059–6063. 10.1073/pnas.93.12.6059 8650219 PMC39188

[B3] Amato S. M. Orman M. A. Brynildsen M. P. (2013). Metabolic control of persister formation in *Escherichia coli*. Mol. Cell 50, 475–487. 10.1016/j.molcel.2013.04.002 23665232

[B4] Bibi-Triki S. Li de la Sierra-Gallay I. Lazar N. Leroy A. Van Tilbeurgh H. Sebbane F.. (2014). Functional and structural analysis of HicA3-HicB3, a novel toxin-antitoxin system of *Yersinia pestis*. J. Bacteriol. 196, 3712–3723. 10.1128/JB.01932-14 25112480 PMC4248797

[B5] Blower T. R. Fineran P. C. Johnson M. J. Toth I. K. Humphreys D. P. Salmond G. P. C. (2009). Mutagenesis and functional characterization of the RNA and protein components of the toxIN abortive infection and toxin-antitoxin locus of *Erwinia*. J. Bacteriol. 191, 6029–6039. 10.1128/JB.00720-09 19633081 PMC2747886

[B6] Blower T. R. Pei X. Y. Short F. L. Fineran P. C. Humphreys D. P. Luisi B. F.. (2011). A processed noncoding RNA regulates an altruistic bacterial antiviral system. Nat. Struct. Mol. Biol. 18, 185–190. 10.1038/nsmb.1981 21240270 PMC4612426

[B7] Boynton T. O. McMurry J. L. Shimkets L. J. (2013). Characterization of *Myxococcus xanthus* MazF and implications for a new point of regulation. Mol. Microbiol. 87, 1267–1276. 10.1111/mmi.12165 23369184 PMC3596438

[B8] Brown B. L. Grigoriu S. Kim Y. Arruda J. M. Davenport A. Wood T. K.. (2009). Three dimensional structure of the MqsR:MqsA complex: a novel toxin:antitoxin pair comprised of a toxin homologous to RelE and an antitoxin with unique properties. PLoS Pathog. 5:e1000706. 10.1371/journal.ppat.1000706 20041169 PMC2791442

[B9] Brown B. L. Lord D. M. Grigoriu S. Peti W. Page R. (2013). The *E. coli* toxin MqsR destabilizes the transcriptional repression complex formed between the antitoxin MqsA and the mqsRA operon promoter. J. Biol. Chem. 288, 1286–1294. 10.1074/jbc.M112.421008 23172222 PMC3543012

[B10] Cao Z. Casabona M. G. Kneuper H. Chalmers J. D. Palmer T. (2016). The type VII secretion system of *Staphylococcus aureus* secretes a nuclease toxin that targets competitor bacteria. Nat. Microbiol. 2:16183. 10.1038/nmicrobiol.2016.183 27723728 PMC5325307

[B11] Cheng H.-Y. Soo V. W. C. Islam S. McAnulty M. J. Benedik M. J. Wood T. K. (2014). Toxin GhoT of the GhoT/GhoS toxin/antitoxin system damages the cell membrane to reduce adenosine triphosphate and to reduce growth under stress. Environ. Microbiol. 16, 1741–1754. 10.1111/1462-2920.12373 24373067

[B12] Darwin C. (1859). On the Origin of Species. London, UK: John Murray.

[B13] Dörr T. Vulić M. Lewis K. (2010). Ciprofloxacin causes persister formation by inducing the TisB toxin in *Escherichia coli*. PLoS Biol. 8:e1000317. 10.1371/journal.pbio.1000317 20186264 PMC2826370

[B14] Dy R. L. Przybilski R. Semeijn K. Salmond G. P. C. Fineran P. C. (2014). A widespread bacteriophage abortive infection system functions through a Type IV toxin–antitoxin mechanism. Nucleic Acids Res. 42, 4590–4605. 10.1093/nar/gkt1419 24465005 PMC3985639

[B15] Ehrenberg M. Sverredal A. (1995). A model for copy number control of the plasmid R1. J. Mol. Biol. 246, 472–485. 10.1006/jmbi.1994.0099 7877168

[B16] Engelberg-Kulka H. Glaser G. (1999). Addiction modules and programmed cell death and antideath in bacterial cultures. Annu. Rev. Microbiol. 53, 43–70. 10.1146/annurev.micro.53.1.43 10547685

[B17] Fineran P. C. Blower T. R. Foulds I. J. Humphreys D. P. Lilley K. S. Salmond G. P. C. (2009). The phage abortive infection system, ToxIN, functions as a protein–RNA toxin–antitoxin pair. Proc. Natl. Acad. Sci. U.S.A. 106, 894–899. 10.1073/pnas.0808832106 19124776 PMC2630095

[B18] Gao Y. Chen K. Zhang B. Li X. Chen L. Li Y.. (2010). Antioxidant and free radical-scavenging activity of the extracellular death factor in *Escherichia coli*. Peptides 31, 1821–1825. 10.1016/j.peptides.2010.06.034 20621143

[B19] Gerdes K. Bech F. W. Jorgensen S. T. Lobner-Olesen A. Rasmussen P. B. Atlung T.. (1986a). Mechanism of postsegregational killing by the *hok* gene product of the *parB* System of Plasmid R1 and its homology with the RelF gene product of the *E. coli relB* operon. Eur. Mol. Biol. Org. J. 5, 2023–2029. 3019679 10.1002/j.1460-2075.1986.tb04459.x PMC1167073

[B20] Gerdes K. Rasmussen P. B. Molin S. (1986b). Unique type of plasmid maintenance function: postsegregational killing of plasmid-free cells. Proc. Natl. Acad. Sci. U.S.A. 83, 3116–3120. 10.1073/pnas.83.10.3116 3517851 PMC323463

[B21] Guo Y. Quiroga C. Chen Q. McAnulty M. J. Benedik M. J. Wood T. K.. (2014). RalR (a DNase) and RalA (a small RNA) form a type I toxin–antitoxin system in *Escherichia coli*. Nucleic Acids Res. 42, 6448–6462. 10.1093/nar/gku279 24748661 PMC4041452

[B22] Harms A. Brodersen D. E. Mitarai N. Gerdes K. (2018). Toxins, targets, and triggers: an overview of toxin-antitoxin biology. Mol. Cell. [Epub ahead of print]. 10.1016/j.molcel.2018.01.003 29398446

[B23] Hazan R. Engelberg-Kulka H. (2004). *Escherichia coli mazEF*-mediated cell death as a defense mechanism that inhibits the spread of phage P1. Mol. Genet. Genomics 272, 227–234. 10.1007/s00438-004-1048-y 15316771

[B24] Hu Y. Benedik M. J. Wood T. K. (2012). Antitoxin DinJ influences the general stress response through transcript stabilizer CspE. Environ. Microbiol. 14, 669–679. 10.1111/j.1462-2920.2011.02618.x 22026739 PMC7261204

[B25] Kim J.-S. Wood T. K. (2016). Persistent persister misperceptions. Front. Microbiol. 7:2134. 10.3389/fmicb.2016.02134 28082974 PMC5187198

[B26] Kim J.-S. Wood T. K. (2017). Tolerant, growing cells from nutrient shifts are not persister cells. MBio 8:e00354-17. 10.1128/mBio.00354-17 28420737 PMC5395667

[B27] Kim Y. Wood T. K. (2010). Toxins Hha and CspD and small RNA regulator Hfq are involved in persister cell formation through MqsR in *Escherichia coli*. Biochem. Biophys. Res. Commun. 391, 209–213. 10.1016/j.bbrc.2009.11.033 19909729 PMC2812665

[B28] Kolodkin-Gal I. Hazan R. Gaathon A. Carmeli S. Engelberg-Kulka H. (2007). A linear pentapeptide is a quorum-sensing factor required for *mazEF*-mediated cell death in *Escherichia coli*. Science 318, 652–655. 10.1126/science.1147248 17962566

[B29] Kumar S. Kolodkin-Gal I. Vesper O. Alam N. Schueler-Furman O. Moll I.. (2016). *Escherichia coli* quorum-sensing EDF, a peptide generated by novel multiple distinct mechanisms and regulated by trans-translation. mBio 7:e02034-15. 10.1128/mBio.02034-15 26814184 PMC4742708

[B30] Kwan B. W. Lord D. M. Peti W. Page R. Benedik M. J. Wood T. K. (2015a). The MqsR/MqsA Toxin/Antitoxin system protects *Escherichia coli* during bile acid stress. Environ. Microbiol. 17, 3168–3181. 10.1111/1462-2920.12749 25534751

[B31] Kwan B. W. Osbourne D. O. Hu Y. Benedik M. J. Wood T. K. (2015b). Phosphodiesterase DosP increases persistence by reducing cAMP which reduces the signal indole. Biotechnol. Bioeng. 112, 588–600. 10.1002/bit.25456 25219496

[B32] Lee B. Holkenbrink C. Treuner-Lange A. Higgs P. I. (2012). *Myxococcus xanthus* developmental cell fate production: heterogeneous accumulation of developmental regulatory proteins and reexamination of the role of MazF in developmental lysis. J. Bacteriol. 194, 3058–3068. 10.1128/JB.06756-11 22493014 PMC3370845

[B33] Lee J.-H. Wood T. K. Lee J. (2015). Roles of indole as an interspecies and interkingdom signaling molecule. Trends Microbiol. 23, 707–718. 10.1016/j.tim.2015.08.001 26439294

[B34] Marimon O. Teixeira J. M. C. Cordeiro T. N. Soo V. W. C. Wood T. L. Mayzel M.. (2016). An oxygen-sensitive toxin–antitoxin system. Nat. Commun. 7:13634. 10.1038/ncomms13634 27929062 PMC5155140

[B35] Masuda H. Tan Q. Awano N. Wu K.-P. Inouye M. (2012). YeeU enhances the bundling of cytoskeletal polymers of MreB and FtsZ, antagonizing the CbtA (YeeV) toxicity in *Escherichia coli*. Mol. Microbiol. 84, 979–989. 10.1111/j.1365-2958.2012.08068.x 22515815

[B36] Nariya H. Inouye M. (2008). MazF, an mRNA interferase, mediates programmed cell death during multicellular *Myxococcus* development. Cell 132, 55–66. 10.1016/j.cell.2007.11.044 18191220

[B37] Ogura T. Hiraga S. (1983). Mini-F plasmid genes that couple host cell division to plasmid proliferation. Proc. Natl. Acad. Sci. U.S.A. 80, 4784–4788. 10.1073/pnas.80.15.4784 6308648 PMC384129

[B38] Otsuka Y. Yonesaki T. (2012). Dmd of bacteriophage T4 functions as an antitoxin against *Escherichia coli* LsoA and RnlA toxins. Mol. Microbiol. 83, 669–681. 10.1111/j.1365-2958.2012.07975.x 22403819

[B39] Page R. Peti W. (2016). Toxin-antitoxin systems in bacterial growth arrest and persistence. Nat. Chem. Biol. 12, 208–214. 10.1038/nchembio.2044 26991085

[B40] Pandey D. P. Gerdes K. (2005). Toxin–antitoxin loci are highly abundant in free-living but lost from host-associated prokaryotes. Nucleic Acid Res. 33, 966–976. 10.1093/nar/gki201 15718296 PMC549392

[B41] Pecota D. C. Wood T. K. (1996). Exclusion of T4 phage by the *hok/sok* killer locus from plasmid R1. J. Bacteriol. 178, 2044–2050. 10.1128/jb.178.7.2044-2050.1996 8606182 PMC177903

[B42] Pedersen K. Christensen S. Gerdes K. (2002). Rapid induction and reversal of a bacteriostatic condition by controlled expression of toxins and antitoxins. Mol. Microbiol. 45, 501–510. 10.1046/j.1365-2958.2002.03027.x 12123459

[B43] Pereira C. S. Thompson J. A. Xavier K. B. (2013). AI-2-mediated signalling in bacteria. FEMS Microbiol. Rev. 37, 156–181. 10.1111/j.1574-6976.2012.00345.x 22712853

[B44] Ren D. Bedzyk L. A. Thomas S. M. Ye R. W. Wood T. K. (2004). Gene expression in *Escherichia coli* biofilms. Appl. Microbiol. Biotechnol. 64, 515–524. 10.1007/s00253-003-1517-y 14727089

[B45] Rendueles O. Beloin C. Latour-Lambert P. Ghigo J.-M. (2014). A new biofilm-associated colicin with increased efficiency against biofilm bacteria. ISME J. 8, 1275–1288. 10.1038/ismej.2013.238 24451204 PMC4030232

[B46] Ruangprasert A. Maehigashi T. Miles S. J. Giridharan N. Liu J. X. Dunham C. M. (2014). Mechanisms of toxin inhibition and transcriptional repression by *Escherichia coli* DinJ-YafQ. J. Biol. Chem. 289, 20559–20569. 10.1074/jbc.M114.573006 24898247 PMC4110269

[B47] Santiago A. D. Mendes J. S. Santos C. A. Toledo M. A. Beloti L. L. Crucello A.. (2016). The antitoxin protein of a toxin-antitoxin system from *Xylella fastidiosa* is secreted via outer membrane vesicles. Front. Microbiol. 7:2030. 10.3389/fmicb.2016.02030 28066356 PMC5167779

[B48] Sat B. Hazan R. Fisher T. Khaner H. Glaser G. Engelberg-Kulka H. (2001). Programmed cell death in *Escherichia coli*: some antibiotics can trigger mazEFLethality. J. Bacteriol. 183, 2041–2045. 10.1128/JB.183.6.2041-2045.2001 11222603 PMC95100

[B49] Sberro H. Leavitt A. Kiro R. Koh E. Peleg Y. Qimron U.. (2013). Discovery of functional Toxin/Antitoxin systems in bacteria by shotgun cloning. Mol. Cell 50, 136–148. 10.1016/j.molcel.2013.02.002 23478446 PMC3644417

[B50] Schmidt T. M. (2012). Bacteria battling for survival, in Microbes and Evolution, eds Kolter R. Maloy S. (Washington, DC: American Society of Microbiology), 59–64.

[B51] Schureck M. A. Maehigashi T. Miles S. J. Marquez J. Cho S. E. Erdman R.. (2014). Structure of the *Proteus vulgaris* HigB-(HigA)2-HigB toxin-antitoxin complex. J. Biol. Chem. 289, 1060–1070. 10.1074/jbc.M113.512095 24257752 PMC3887174

[B52] Shidore T. Broeckling C. D. Kirkwood J. S. Long J. J. Miao J. Zhao B.. (2017). The effector AvrRxo1 phosphorylates NAD in planta. PLOS Pathogens 13:e1006442. 10.1371/journal.ppat.1006442 28628666 PMC5491322

[B53] Soo V. W. C. Wood T. K. (2013). Antitoxin MqsA represses curli formation through the master biofilm regulator CsgD. Sci. Rep. 3:3186. 10.1038/srep03186 24212724 PMC4894380

[B54] Tsilibaris V. Maenhaut-Michel G. Mine N. Van Melderen L. (2007). What is the benefit to *Escherichia coli* of having multiple toxin-antitoxin systems in its genome? J. Bacteriol. 189, 6101–6108. 10.1128/JB.00527-07 17513477 PMC1951899

[B55] Wang X. Kim Y. Hong S. H. Ma Q. Brown B. L. Pu M.. (2011). Antitoxin MqsA helps mediate the bacterial general stress response. Nat. Chem. Biol. 7, 359–366. 10.1038/nchembio.560 21516113 PMC3097263

[B56] Wang X. Lord D. M. Cheng H. Y. Osbourne D. O. Hong S. H. Sanchez-Torres V.. (2012). A new type V toxin-antitoxin system where mRNA for toxin GhoT is cleaved by antitoxin GhoS. Nat. Chem. Biol. 8, 855–861. 10.1038/nchembio.1062 22941047 PMC3514572

[B57] Wang X. Lord D. M. Hong S. H. Peti W. Benedik M. J. Page R.. (2013). Type II toxin/antitoxin MqsR/MqsA controls type V toxin/antitoxin GhoT/GhoS. Environ. Microbiol. 15, 1734–1744. 10.1111/1462-2920.12063 23289863 PMC3620836

[B58] Wang X. Wood T. K. (2011). Toxin-antitoxin systems influence biofilm and persister cell formation and the general stress response. Appl. Environ. Microbiol. 77, 5577–5583. 10.1128/AEM.05068-11 21685157 PMC3165247

[B59] Wen W. Liu B. Xue L. Zhu Z. Niu L. Sun B. (2018). Autoregulation and virulence control by the toxin-antitoxin system SavRS in *Staphylococcus aureus*. Infect. Immun. [Epub ahead of print]. 10.1128/IAI.00032-18 29440365 PMC5913840

[B60] Yamaguchi Y. Park J.-H. Inouye M. (2009). MqsR, a crucial regulator for quorum sensing and biofilm formation, is a GCU-specific mRNA interferase in *Escherichia coli*. J. Biol. Chem. 284, 28746–28753. 10.1074/jbc.M109.032904 19690171 PMC2781420

[B61] Yan Z. Li G. Gao Y. Zhai W. Qi Y. Zhai M. (2015). The extracellular death factor (EDF) protects *Escherichia coli* by scavenging hydroxyl radicals induced by bactericidal antibiotics. Springerplus 4:182. 10.1186/s40064-015-0968-9 25932369 PMC4411399

[B62] Zhang Y. Zhang J. Hoeflich K. P. Ikura M. Qing G. Inouye M. (2003). MazF cleaves cellular mRNAs specifically at ACA to block protein synthesis in *Escherichia coli*. Mol. Cell 12, 913–923. 10.1016/S1097-2765(03)00402-7 14580342

